# Concurrent Occurrence of Ear Tag With Posterior Talon Cusp, Fissured Tongue, and Ankyloglossia: A Case Report

**DOI:** 10.7759/cureus.42095

**Published:** 2023-07-18

**Authors:** Mirnalini Sundaresan, Ramachandra Venkatesha reddy, Vasu Sridharrao, Saramma Mathew Fenn, Karthik Rajaram Mohan

**Affiliations:** 1 Oral Medicine and Radiology, Vinayaka Mission's Sankarachariyar Dental College, Vinayaka Mission's Research Foundation (Deemed to be University), Salem, IND

**Keywords:** branchial arch syndrome, branchial arch, hemifacial microsomia, goldenhar syndrome, ear

## Abstract

Ear tags or accessory auricles are branchial cleft remnants that clinically appear as asymptomatic nodules or papules in the preauricular region. They occur in various syndromes affecting the first and branchial arches during embryogenesis. The presence of an ear tag can have a psychological impact on one's life due to its unesthetic appearance, thereby affecting their quality of life. Talon cusp usually occurs in the maxillary central or lateral incisor. A fissured tongue or cerebriform tongue is characterized by the presence of horizontal or vertical grooves, usually affecting the dorsum of the tongue. Ankyloglossia or tongue-tie is a developmental anomaly in which the lingual frenum is abnormally attached to the ventral surface of the tongue. It can cause difficulties in breastfeeding in infants and in the pronunciation of certain vowels in adults. The concurrent occurrence of the ear tag along with the talon cusp in the mandibular second molar has not been reported in previous literature. We present a unique case of a 24-year-old non-syndromic individual with the concurrent occurrence of the ear tag along with a rare clinical occurrence of talon cusp in the mandibular second molar, fissured tongue, and ankyloglossia.

## Introduction

The ear tag is a rudimentary skin tag that often contains ear tissue, including a cartilage core anterior to the auricle [1]. Ear tags occur due to the failure of the fusion of six auricular hillocks that form during external ear development during birth [2]. Ear tags are asymptomatic, and affected patients may complain of a cosmetic problem [2]. Ear tags occur in first and second branchial arch syndromes such as Goldenhar syndrome and hemifacial microsomia. Ear tags can arise in the posterior helix and lobule junction or between the superior and posterior helices since the cartilage is more prone to in utero and ex utero deformational forces [2]. Ankyloglossia results from an X-linked cleft palate syndrome caused by a mutation in the *TBX22* gene. Ankyloglossia or tongue-tie results from a short lingual frenum attached to the ventral surface near the tip of the tongue. This condition can interfere with breastfeeding in infants and cause difficulties in pronouncing words such as t, sh, and th. The prevalence of ankyloglossia varies from 1% to 10% of the population [3]. Talon cusp is a rare developmental anomaly affecting the teeth, resulting in talon-shaped cusp-like structures. Talon cusp can hinder occlusion and is more prone to plaque accumulation, resulting in dental caries and underlying pulp infections since a core of pulp tissue extends close to the pulp chamber, which results in easy insults from chemical agents or restorative materials [4].

## Case presentation

**Figure 1:**
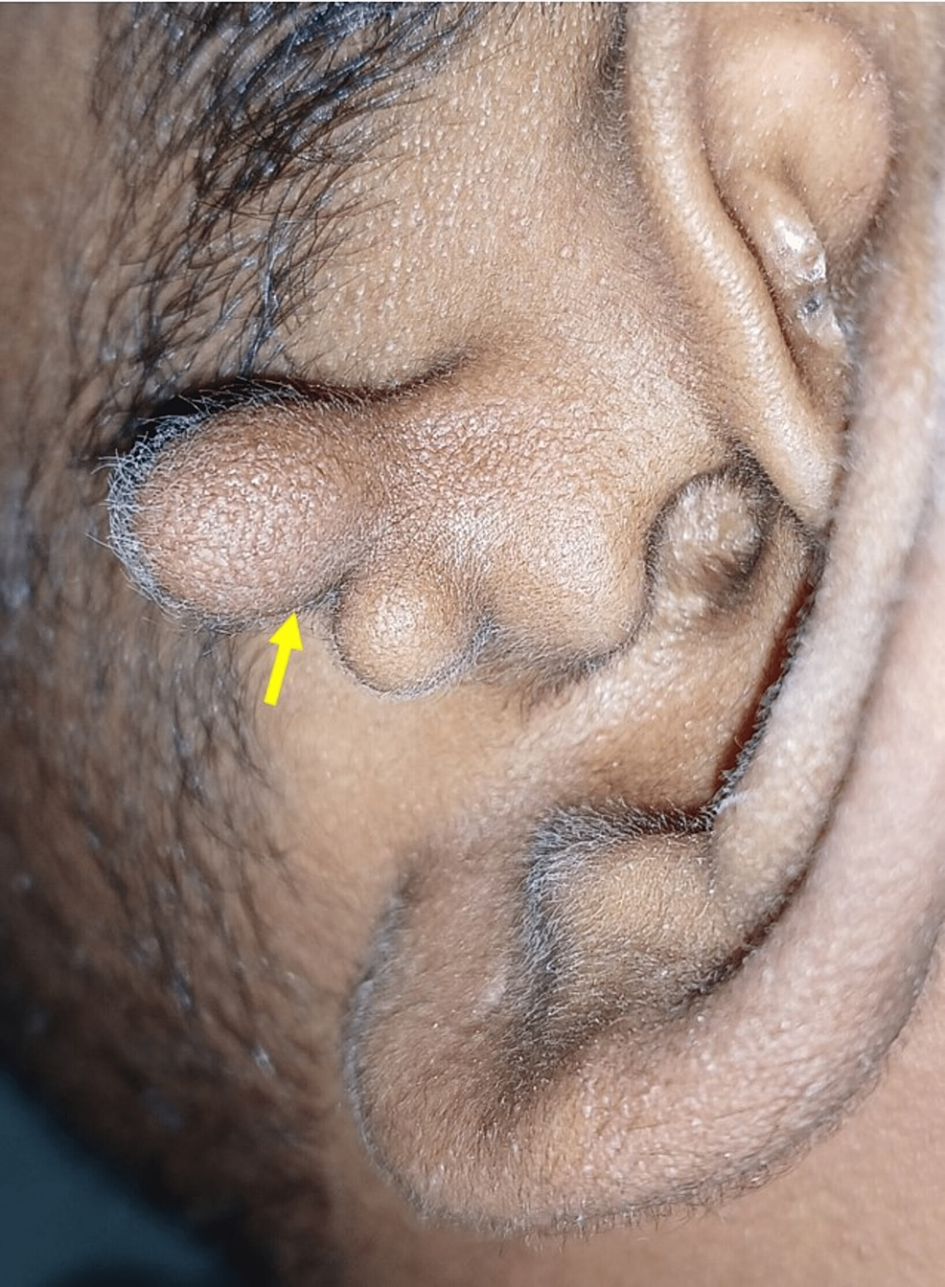
Clinical photograph showing a dumbbell-like structure in the left preauricular region.

A 24-year-old male reported to the dental outpatient department for a routine checkup. On extraoral examination, a growth was seen in his left preauricular region. On inspection, the bump was shaped like a bifid-dumbbell-like structure with a stalk (Figure 1).

The growth on palpation is non-tender, soft in consistency, compressible, and reducible with a pedunculated stalk (Video 1).

**Video 1:** Clinical examination of the ear tag.

**Figure 2:**
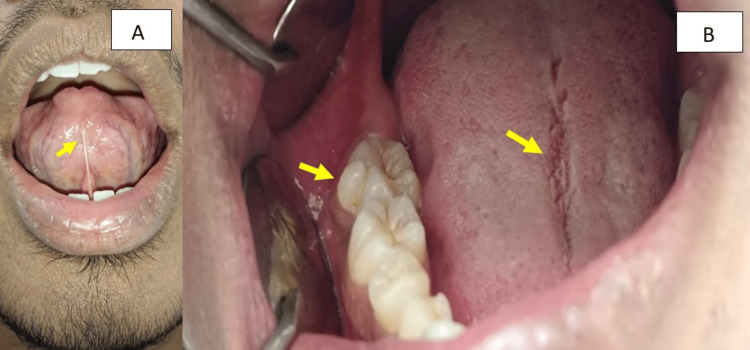
Intraoral examination revealed (A) ankyloglossia (tongue-tie) and (B) talon cusp on the right mandibular second molar and fissured tongue (central longitudinal variety) on the dorsum of the tongue.

Intraoral soft tissue examination revealed ankyloglossia (tongue-tie), a rudimentary cusp resembling a talon cusp on the right second molar region, and a fissured tongue with a central longitudinal variety (Figure 2A, B). 

The differential diagnosis for ear tags includes pretragal ectopias or striated muscle hamartoma [2]. Pretragal ectopias often appear helix-like, and such cases are referred to as polyotia [2]. The peculiarity seen in our case was that ear tags usually lack hair follicles, whereas fine hairs are seen on the ear tags' surface. Talon cusp, normally seen on the anterior maxillary teeth (maxillary central and lateral incisors), occurred on the mandibular posterior teeth, namely the buccal surface of the mandibular second molar. In our case, the treatment done for talon cusp is professionally applied topical application of 5% sodium fluoride varnish (Voco Profluorid Varnish, Voco America Inc, Fort Mill, South Carolina, United States) after enameloplasty to restore occlusion to the retruded contact position.

## Discussion

Ear tags/accessory auricles/branchial cleft remnant

**Figure 3:**
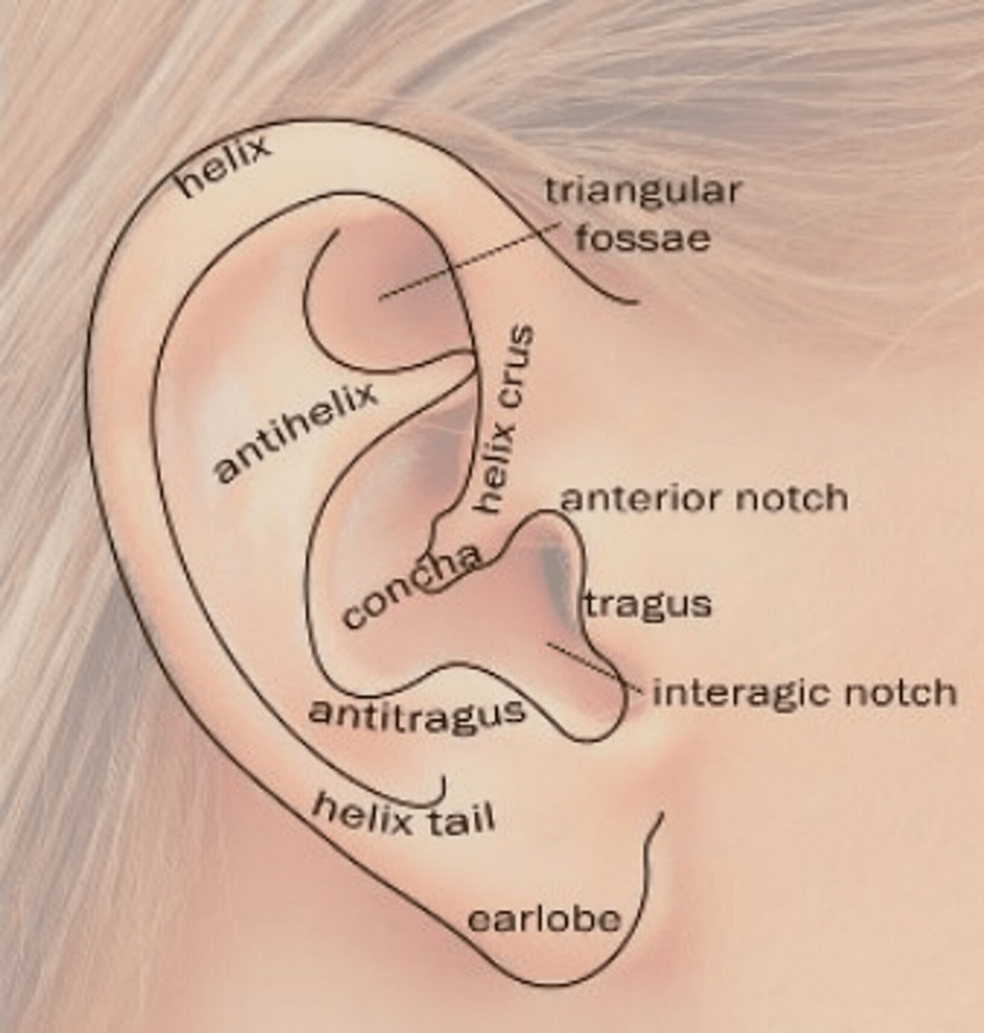
The topographic surface anatomy of the ear. Image courtesy: https://oxfordmedicaleducation.com/clinical-examinations/ear-examination/

Ear tags are benign growths containing skin and ear tissue, including a core of cartilage located just anterior to the auricle [1]. They are known by other names such as accessory auricles, supernumerary tragus, preauricular appendages, branchial cleft remnants, accessory tragi, heterotrophic tragi, accessory tags, rudimentary ear, polyotia, or supernumerary pinna [1]. Ear tags are rudimentary tags of ear tissue, typically located in front of the ear (preauricular) region; hence, they are also called preauricular tags [2]. They are most often unilateral but can be rarely bilateral and multiple [2]. Clinically, they appear as skin-colored nodules or papules in the preauricular region at birth [2]. It is a common benign anomaly first reported by Birket in 1858 [2]. The ear tag is the most common among developmental ear anomalies, with an estimated prevalence rate of 1.7:1000 [2]. The surface anatomy of the ear is depicted in Figure 3.

The site of preauricular appendages appears to be very constant: between the crus helicis and the upper part of the tragus, or from the tragus as a center, they may extend upward and forward toward the temporal region, downward toward the cheek, or between the anti-tragus and the lower part of the tragus. It is, therefore, clear that the line of the election of preauricular appendages lies just anterior to preauricular fistulae [3].

Ear tags are formed when the auricular hillocks fuse incompletely, resulting in an additional appendage in front of the ear [4]. Ear tags are composed of fat, skin, and cartilage occasionally. Ear tags are present from birth [4]. Pretragal ectopias or polyotia clinically mimic ear tags but are usually helical in shape in the preauricular region [5]. Ear tags occur in genetic syndromes such as hemifacial microsomia, Goldenhar syndrome, Haberland syndrome, Delleman syndrome, Townes-Brocks syndrome, and first and second branchial arch syndromes [6]. They are also associated with hearing loss [4]. Hence, people with ear tags must have audiometry tests to rule out hearing loss [6]. The treatment of ear tags depends on whether they contain rudimentary cartilage or a core of cartilage stalk [6]. Smart clipping devices can remove ear tags with rudimentary cartilage devices without general anesthesia [6]. Ear tags with cartilage stalk require surgical excision under general anesthesia [6]. Ear tags containing cartilage must be carefully excised to avoid surgical scars or bumps [7]. Adequate care must be taken during surgery to prevent injury and paralysis to the facial nerve, which is nearby about 4 mm in the preauricular region [7].

Talon cusp

The excitation of the dental organ during the morpho-differentiation stage, along with genetic and environmental influences, is essential for forming the talon cusp. Talon cusp can occur in both primary and permanent dentition [8]. The talon cusp clinically appears as a talon-shaped cusp-like structure extending from the incisal edge of the lingual aspect of maxillary and mandibular anterior teeth and projecting to the level of the cingulum [9]. Some talon cusp also occurs on the labial surface of the anterior maxillary teeth [10]. The incidence of the talon cusp is 0.001% [10]. The prevalence of talon cusp in India has been reported as 2.95% in North India and 0.58% in South India [10]. Talon cusp is reported in patients with Rubinstein-Taybi syndrome and teeth with cleft palate syndromes, Ellis-van Creveld syndrome, impacted teeth, compound odontoma, and taurodontism [10]. An unusual variant of the Talon cusp is called a ternion cusp, clinically in which three very well-defined mamelon-like cusps occur on the palatal side of the permanent maxillary central incisor [10].

Classification of Talon Cusp

The classification of the talon cusp is presented in Table 1 [5].

**Table 1:** Classification of talon cusp. F: facial; L/P: lingual/palatal; FL/P: facial and lingual/palatal; U: unilateral; BL: bilateral.

Type of talon cusp	Name of talon cusp	Clinical features
Type 1	Talon	A morphologically well-delineated additional cusp that prominently projects from the palatal surface usually or facial surface rarely in primary or permanent maxillary central lateral or canine tooth and extends at least half the distance from the incisal edge to the cementoenamel junction.
Type 2	Semi talon	An additional cusp of a millimeter or more extending less than half the distance from the incisal edge to the cementoenamel junction. It may stand away from the rest of the crown or blend with the palatal surface.
Type 3	Trace talon	Prominent or enlarged cingula and its variations, that is, bifid, tubercle-like, or conical.
Based on the surface involved	F	
	L/P	
	FL/P	
Comprehensive integrated classification system of talon cusp	Teeth involved in Fédération Dentaire Internationale system	
	U or BL	
	Degree of cusp formation ranging from TI to TIII	
	Surface involved ranging from F to FL/P	

Talon cusps interfere with occlusion, cause gingival irritation from the buildup of bacterial plaque, make the teeth more caries-prone, result in tooth fracture due to abnormal occlusal forces, and cause pain around the temporomandibular joint [10].

Ankyloglossia or tongue-tie

Ankyloglossia or tongue-tie is a short lingual frenum attached to the ventral surface near the tip of the tongue and results in difficulty in the pronunciation of certain words, such as the combination of letters: t, r, l, d, n, z, sh, and th [11]. The treatment of ankyloglossia includes frenotomy under local anesthesia or frenuloplasty under general anesthesia [11]. Myofunctional therapy has promising effectiveness in treating ankyloglossia [11]. Ankyloglossia can also be found in many congenital syndromes, such as Beckwith-Wiedemann, Simosa, Ehlers-Danlos, Simpson-Golabi-Behmel, oral-facial digital syndromes, Opitz, and X-linked cleft palate, and in newborns from cocaine-addicted mothers [11]. Lingual laser frenotomy reduces complications such as less bleeding, rapid healing, intraoperative pain, and complications [12]. Ankyloglossia can lead to dental problems, such as spacing between the mandibular incisors [12,13].

Fissured tongue/scrotal tongue/plicated tongue/grooved tongue/lingua plicata/lingua fissurata

A fissured tongue is characterized by grooves that vary in depth along the dorsal aspects of the tongue [14]. The etiology of the fissured tongue is unclear [14]. The fissured tongue is usually asymptomatic unless food debris gets entrapped in it, leading to an occasional burning sensation on the tongue and halitosis [14]. The fissured tongue is generally diagnosed during a routine intraoral clinical examination by the dentist. Fissured tongue occurs in patients with Melkersson-Rosenthal syndrome, Down syndrome, benign migratory glossitis (erythema migrans or geographic tongue), pernicious anemia, Sjögren's syndrome, psoriasis, macroglossia, oral-facial-digital syndrome type I, Pierre Robin syndrome, acromegaly, Coffin-Lowry syndrome, Fraser syndrome, Sjogren's syndrome, Maroteaux-Lamy syndrome, Mohr syndrome, and ectrodactyly-ectodermal dysplasia-cleft syndrome [14,15].

The classification of the fissured tongue is described in Table 2 [15].

**Table 2:** Classification of the fissured tongue.

Based on the pattern of tongue fissures	Central longitudinal pattern	Vertical fissure that extends along the midline of the superior surface of the tongue
	Central transverse pattern	Horizontal fissure/fissures that are crossing the midline of the tongue
	Lateral longitudinal pattern	Vertical fissure/fissures running laterally to the midline of the tongue
	Branching pattern	Transverse fissures that extend from the midline longitudinal fissure (branching tree appearance)
	Diffuse pattern	Fissures that are widely distributed across the dorsal surface of the tongue
Based on the number of tongue fissures	Mild	Tongue fissures that are 1–3 in number
	Moderate	Tongue with >3 fissures
	Severe	Tongue with >10 fissures
Based on the presence or absence of analogous symptoms such as burning sensation		Absence of burning sensation
		Presence of burning sensation

Sudarshan et al. stated that fissured tongue has a polygenic mode of inheritance, and about 76.9% (196 of the total examined 387 subjects (235 males and 152 females)) had a variety of central longitudinal patterns. In his study, only one case of ankyloglossia was associated with fissured tongue [16].

## Conclusions

The presence of ear tags indicates a developmental defect from birth. A thorough clinical review of systems, such as hearing loss and renal or nervous system examination, should be evaluated when such an ear tag is diagnosed. Dentists must pay attention to the occurrence of other developmental anomalies that can occur with ear tags. A thorough dental examination must also look for developmental anomalies such as ankyloglossia and talon cusp, which can occur concurrently with ear tags. Ankyloglossia may hinder speech function, and the tongue's protrusive movement results in difficulty pronouncing certain words. Talon cusp can interfere with occlusion and cause temporomandibular joint pain. It can accumulate dental plaque, making teeth more caries-prone, susceptible to tooth decay, and causing dental pain. If not treated, such developmental anomalies can affect one's self-esteem, create a psychological impact, and affect a patient's quality of life.
